# The Cytolethal Distending Toxin Subunit CdtB of *Helicobacter hepaticus* Promotes Senescence and Endoreplication in Xenograft Mouse Models of Hepatic and Intestinal Cell Lines

**DOI:** 10.3389/fcimb.2017.00268

**Published:** 2017-06-30

**Authors:** Christelle Péré-Védrenne, Martina Prochazkova-Carlotti, Benoit Rousseau, Wencan He, Lucie Chambonnier, Elodie Sifré, Alice Buissonnière, Pierre Dubus, Francis Mégraud, Christine Varon, Armelle Ménard

**Affiliations:** ^1^Institut National de la Santé et de la Recherche Médicale, UMR1053 Bordeaux Research in Translational Oncology, BaRITOnBordeaux, France; ^2^UMR1053 Bordeaux Research in Translational Oncology, BaRITOn, Université de BordeauxBordeaux, France; ^3^Service Commun des Animaleries, Université de BordeauxBordeaux, France; ^4^CHU Hôpitaux de Bordeaux, Pôle Biologie et Pathologie, Service de Biologie des TumeursBordeaux, France

**Keywords:** *Helicobacter hepaticus*, cytolethal distending toxin, endoreplication, apoptosis, senescence, mitosis

## Abstract

Cytolethal distending toxins (CDTs) are common among pathogenic bacteria of the human and animal microbiota. CDTs exert cytopathic effets, via their active CdtB subunit. No clear description of those cytopathic effects has been reported at the cellular level in the target organs *in vivo*. In the present study, xenograft mouse models of colon and liver cell lines were set up to study the effects of the CdtB subunit of *Helicobacter hepaticus*. Conditional transgenic cell lines were established, validated *in vitro* and then engrafted into immunodeficient mice. After successful engraftment, mice were treated with doxycyclin to induce the expression of transgenes (red fluorescent protein, CdtB, and mutated CdtB). For both engrafted cell lines, results revealed a delayed tumor growth and a reduced tumor weight in CdtB-expressing tumors compared to controls. CdtB-derived tumors showed γ-H2AX foci formation, an increase in apoptosis, senescence, p21 and Ki-67 nuclear antigen expression. No difference in proliferating cells undergoing mitosis (phospho-histone H3) was observed. CdtB intoxication was also associated with an overexpression of cytokeratins in cells at the invasive front of the tumor as well as an increase in ploidy. All these features are hallmarks of endoreplication, as well as aggressiveness in cancer. These effects were dependent on the histidine residue at position 265 of the CdtB, underlying the importance of this residue in CdtB catalytic activity. Taken together, these data indicate that the CdtB triggers senescence and cell endoreplication leading to giant polyploid cells in these xenograft mouse models.

## Introduction

Cytolethal distending toxins (CDTs) are widely distributed among many pathogenic Gram-negative bacteria including *Escherichia species, Campylobacter* species, *Haemophilus ducreyi, Aggregatibacter actinomycetemcomitans, Salmonella enterica, Shigella* species, *Haemophilus parasuis, Providencia alcalifaciens*, and some enterohepatic *Helicobacter* species. CDT is involved in the severity of the diseases caused by these bacteria and many properties of this toxin support the likelihood of its involvement in cancers (reviewed in Bezine et al., 2014; Faïs et al., 2016). *In vivo*, CDT plays a key role in inflammation and promotes liver carcinogenesis in *Helicobacter hepaticus*-infected mice (Ge et al., 2007) and intestinal carcinogenesis in 129Rag2-deficient mice (Ge et al., 2017). *In vitro*, CDT targets the nucleus where the CdtB subunit displays DNase activity and induces DNA breaks (Bezine et al., 2014, 2016), triggering a DNA repair mechanism, comparable to that induced by ionizing radiation. Chronic exposure to CDT is considered to promote malignant transformation by inducing genomic instability and altering the response to DNA damage (Guidi et al., 2013). CDT intoxication leads to the formation of abnormal nuclei forming mononucleated giant cells with 4–16C DNA content, arrested in G2/M phase, concomitantly with a decline in Cdk1 phosphorylation (Peres et al., 1997; De Rycke et al., 2000; Nougayrede et al., 2001). These latter effects are reminiscent of endoreplication. During endoreplication (or endoreduplication or polytenization), the endocycling cells undergo a complete round of DNA replication (multiple S-phases) in the absence of an intervening mitosis without proliferating or dying, thus leading to elevated nuclear gene content and polyploidy. Endoreplication plays key roles in embryonic development but can also occur in response to certain physiological stresses and contributes to the development of cancer (reviewed in Lee et al., 2009). For cell lines exposed to persistent DNA damage, endoreplication in G2 phase is a well-known mechanism to bypass the G2 permanent arrest leading to cell death (Fox and Duronio, 2013). In the cells that have compromised fidelity of DNA synthesis, these repeated rounds of DNA replication could promote the selection of oncogenic mutations and also prolong the life-span of cancer cells giving rise to daughter cells with different genotypes, some of which might be highly cancerous (Lee et al., 2009; Davoli and de Lange, 2011).

Despite its prevalence, research on CDT has been hampered by difficulties to produce and purify sufficient amounts of the 3 subunits to reconstitute a complete active holotoxin (CdtA, CdtB, and CdtC), CdtB being the active subunit which is toxic for numerous expression systems. As a result, CDT has been little studied. To bypass these technological barriers, we previously implemented a lentiviral-based approach to investigate the effects of the CdtB subunit (Varon et al., 2014; Péré-Védrenne et al., 2016). This system makes it possible to deliver the CdtB of *Helicobacter pullorum* and *H. hepaticus* directly into the cells and to attribute the effects observed, specifically to the active CdtB subunit of the CDT. However, although very useful, this system does not allow the study of longer-term effects of the CdtB subunit or the possibility to conduct experiments requiring large amounts of biological samples, mostly because the CdtB induces G2/M cell cycle arrest *in vitro* (Varon et al., 2014). As constitutive expression of CdtB is incompatible with cell survival and does not allow the establishment of a CdtB-expressing cell line, the use of new lentiviral particles is necessary for each new experiment. To circumvent this issue, we engineered a system for the conditional expression of the CdtB.

In the present study, we report on the construction of lentiviral vectors which were used to establish stable transgenic cell lines that allowed the induction of the conditional expression of *H. hepaticus* CdtB. Once the lentiviral expression systems of CdtB were validated *in vitro*, cells were engrafted into immunodeficient mice. After successful engraftments, the transgene expression was induced for longer-term study and the effects of *H. hepaticus* CdtB were analyzed on tumor growth, apoptosis, senescence, proliferation, differentiation, and ploidy. Similarly the effects of *H. hepaticus* CdtB with a His→Leu mutation at residue 265 (H265L) were also investigated to explore the involvement of the catalytic site of CdtB. Indeed, this residue was shown to be involved in *H. hepaticus* CdtB cytotoxic activity (Avenaud et al., 2004; Péré-Védrenne et al., 2016).

In the context of cancer, the consequences of *in vivo* infections with CDT-secreting bacteria on cancer evolution are poorly understood since it is challenging to identify CDT-intoxicated cells in infected organs. *In vivo* engraftment of cells expressing the toxin in an inducible and stable manner should make it possible to see the effects of CDT in an homogeneous population of CDT stably expressing cells, which is difficult to observe during bacterial infection.

## Materials and methods

Cell lines and culture conditions, *H. hepaticus* strains, reagents and antibodies, the construction of lentiviral plasmids, lentivirus production, histology, immunofluorescence/image analysis, primer design, reverse transcriptase quantitative PCR experiments (RT-qPCR) and statistical analyses are presented in Supplementary Materials and Methods.

### Transduction experiments and establishment of stable transgenic cell lines

Intestinal HT-29 and hepatic Hep3B transgenic cell lines were established by lentiviral transduction (see Supplementary Materials and Methods). Briefly, the pTRIPz lentiviral plasmid with two independent promoters was used: the UBC promoter allowed the constitutive expression of the gene for resistance to puromycin, and the tetracycline response element (TRE) promoter was inducible by tetracycline. The complete *cdtB* sequences of *H. hepaticus* (from the start codon until the codon proximal to STOP codon, GenBank accession numbers: AE017125 or AF163667) fused at their 3′ end to three repeats of the influenza hemagglutinin epitope (HA) (GenBank accession numbers: KT590046 and KT590047) were cloned downstream of the TRE promoter in this plasmid instead of the TurboRFP gene initially present. Cells having the integrated transgene sequence in a transcriptionally silent form were selected in the presence of puromycin (2 μg/ml). When required, the transgene expression was induced in the cells from the tetracycline-inducible promoter by addition of doxycycline (200 ng/ml) to the culture medium and incubation for 72 h.

### Mouse xenografts of HT-29 and Hep3B cells

Successful mice engraftment cannot be achieved with primary cells. Thus, HT-29 and Hep3B carcinoma derived cell lines were used. This study was approved by the Ethics Committee for Animal Care and Experimentation in Bordeaux (“saisine” no. 13126B, Bordeaux, France). All animal experiments were performed in level 2 animal facilities at the University of Bordeaux. Immunodeficient non-obese diabetic (NOD) shi-severe combined immunodeficiency (SCID) interleukin-2Rgammanull (NSG) mice under 2.5% isoflurane anesthesia (Belamont, Piramal Healthcare, Northumberland, UK) were injected subcutaneously into the right shaved flank with 2 × 10^6^ HT-29 cells or 4 × 10^6^ Hep3B cells suspended in 100 μl of culture medium without fetal calf serum. Two engraftment experiments were performed for each cell line, resulting in similar results. The data were thus pooled, enabling to obtain a sufficient number of animals in order to carry out statistical analyses (results presented in **Figure 2**). HT-29 and Hep3B mice groups were each comprised of 40 mice: HT-29 (RFP group *n* = 15, CdtB group *n* = 15, and CdtB-H265L group *n* = 10) and Hep3B (RFP group *n* = 10, CdtB group *n* = 20, and CdtB-H265L group *n* = 10). Tumor growth was monitored 3 times per week by measuring the length (L) and width (W) of the tumor with a caliper, and tumor volume was calculated according to the formula ½ (L × W^2^). When the engrafted HT-29 and Hep3B tumors were visible through the animal's skin and reached approximately 400 and 200 mm^3^, respectively, doxycycline was added constantly at a concentration of 2 mg/ml to the water bottles of all mice. At the end of the experiments, mice were sacrificed by cervical dislocation and tumor weight was determined. Tumors were immediately harvested and divided into 3 pieces. The first piece was directly snap frozen using liquid nitrogen and stored at −80°C. The second was embedded in Tissue-Tek® OCT-compound (Sakura, Labonord, Templemars, France) at −80°C. The third piece was fixed in formalin and embedded in paraffin to be analyzed according our standard protocols (Nguyen et al., 2016).

### Interphase fluorescence *in situ* hybridization (FISH)

Tissue sections (6-μm thick) were prepared from formalin-fixed paraffin-embedded xenografts and FISH experiments were conducted as previously described (Pham-Ledard et al., 2014). Dual-color hybridizations were conducted with labeled probes hybridizing to the band on the short arm of chromosome 5 (chromosome region 5p15.2) (SpectrumGreen probe, Abbott France, Rungis, France) and to the chromosome 15 centromere (SpectrumOrange probe, Abbott France) according to manufacturer's protocol. After post-hybridization washing, slides were mounted with Vectashield antifade medium containing 4′,6-diamidino 2-phenylindole (DAPI) (Vector Laboratories, Laboratoires Eurobio/Abcys, Les Ulis, France) and observed on a Zeiss Axioplan 2 fluorescence microscope (Zeiss, Jena, Germany). Images were captured with a high-resolution camera using Isis software (MetaSystems, Altlussheim, Germany) with subsequent counting of FISH signals, each count being performed on 100 nuclei per mice.

## Results

### Establishment of stable transgenic cell lines

**Figure 1:**
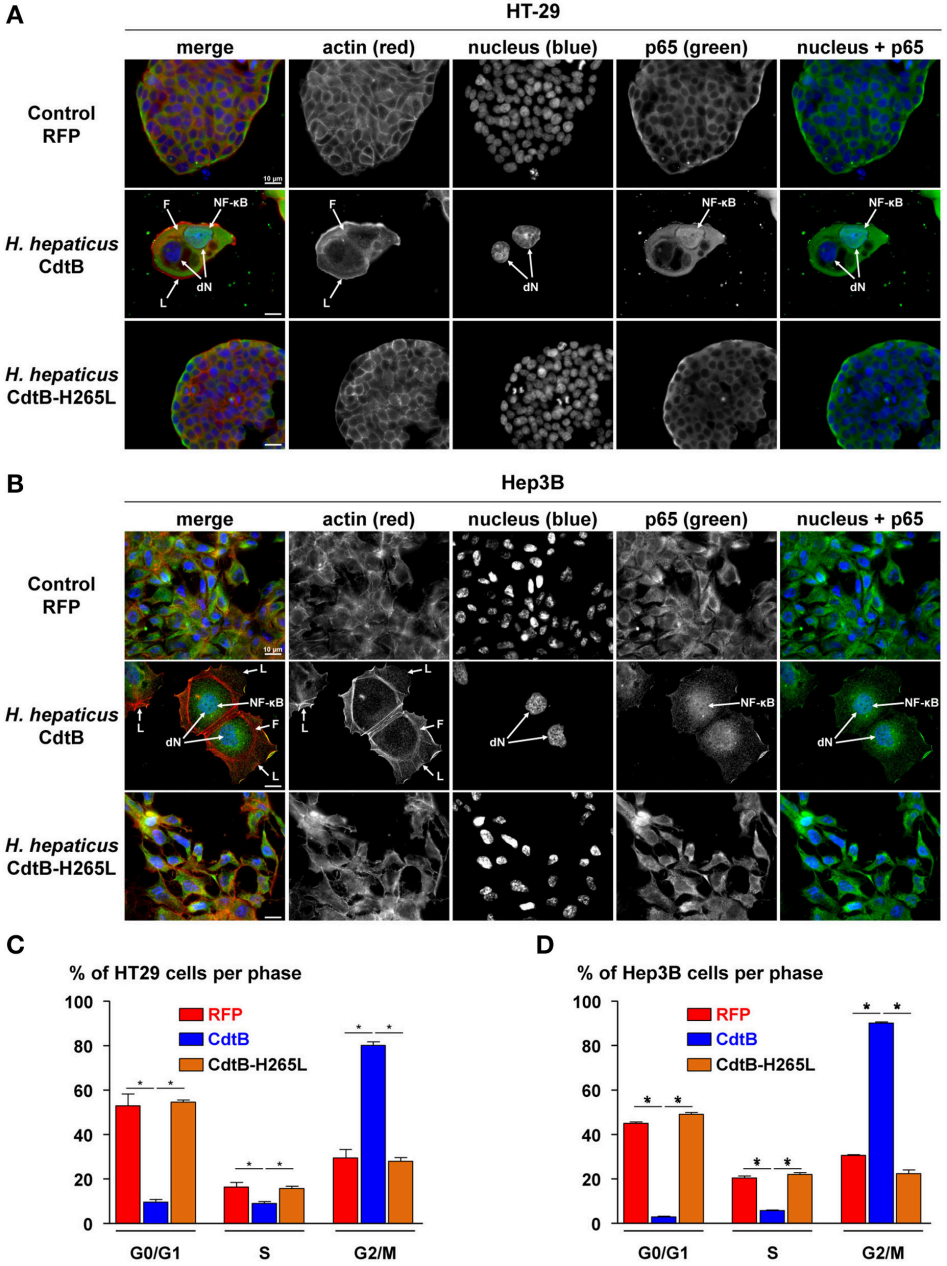
Effects of the CdtB subunit of *Helicobacter hepaticus* CDT on the cytoskeleton and cell cycle of intestinal and hepatic epithelial cell lines. Intestinal HT-29 and hepatic Hep3B transgenic cells were cultivated with doxycycline for 72 h to induce the expression of the control Red Fluorescent Protein (RFP), the CdtB of *H. hepaticus* strain 3B1 (CdtB) or the CdtB of *H. hepaticus* strain 3B1 with the H265L mutation (CdtB-H265L). HT-29 **(A)** and Hep3B **(B)** cells were then processed for fluorescent staining with fluorescent labeled-phalloidin to detect F-actin (infrared), DAPI to counterstain the nucleus (blue) and primary polyclonal antibodies generated against the p65 subunit of the nuclear factor-kappa B (NF-κB) associated with fluorescent labeled-secondary antibodies (green). Fluorescent stainings were observed using traditional widefield fluorescence imaging as previously reported (Péré-Védrenne et al., 2016). Analysis of DNA contents of HT-29 **(C)** and Hep3B **(D)** cells. Cells were fixed and labeled with propidium iodide and analyzed by flow cytometry. Data represent one representative experiment (performed in triplicate) out of 3. ^*^p < 0.05. CdtB, CdtB of *H. hepaticus* strain 3B1; CdtB-H265L, *H. hepaticus* CdtB with H265L mutation; DAPI, 4′,6′-diamidino-2-phenylindol; dN, distended nuclei; F, actin stress fiber structures; L, enlarged lamellipodia; NF-κB, nuclear factor-Kappa B.

*Helicobacter hepaticus* is usually present in the mouse intestine and it colonizes the biliary tract and the liver. Intestinal and liver cells to be engrafted were selected based on their capacity to induce tumors in immunodeficient mice (ATCC recommendations and Devaud et al., 2009; Menard et al., 2010). Epithelial intestinal HT-29 and hepatic Hep3B transgenic cell lines were established by lentiviral transduction, each expressing, on demand, upon doxycycline induction: the TurboRFP, the CdtB of *H. hepaticus* strain 3B1 and the CdtB of *H. hepaticus* strain 3B1 with the His→Leu mutation at residue 265 (H265L) involved in catalytic activity *in vitro* (Avenaud et al., 2004). Both *cdtB* sequences were fused at their 3′ end to three HA repeats. The number of integrated proviral copies, reflecting the number of integrated transgenes, was quantified. HT-29 cells had 3.84 and 3.53 integrated proviral copies harboring *cdtB* and *cdtB*-H265L transgene in their genome, respectively. Hep3B cells had 3.95 and 3.72 integrated proviral copies with *cdtB* and *cdtB*-H265L transgene in their genome, respectively. Regardless of the transduction experiment, the RFP-transduced HT-29 and Hep3B cells had a higher number of proviral copies integrated: 6.87 and 7.66, respectively. The induction of the cytopathogenic effects of the CdtB was verified by immunofluorescence and cell cycle analyses. In both cell lines, the *H. hepaticus* CdtB induced the previously reported effects of the CDT in all of the cells (Figures 1A,B), i.e., a cellular and nuclear enlargement, with the formation of cortical actin-rich large lamellipodia (Varon et al., 2014) and actin stress fibers (Young et al., 2000), as well as the nuclear translocation of NF-κB (Péré-Védrenne et al., 2016). A high increase in the percentage of cells in G2/M phase was observed in response to the CdtB in both cell lines (Figures 1C,D), reflecting the effectiveness of the puromycin selection. Thus, the *H. hepaticus* CdtB induced the expected cellular distending phenotype, actin cytoskeleton remodeling and cell cycle arrest. These effects were not observed in control cells expressing the RFP nor in cells expressing the mutated CdtB-H265L, indicating that the H265L mutation in the catalytic site completely abolished the CdtB-dependent effects, as expected (Avenaud et al., 2004; Péré-Védrenne et al., 2016). Taken together, these results validated the use of these transgenic cell lines established by lentiviral transduction. It should be noted that most of the CdtB expressing cells did not survive beyond 4–5 days *in vitro*.

### The CdtB induced tumor growth delay

**Figure 2:**
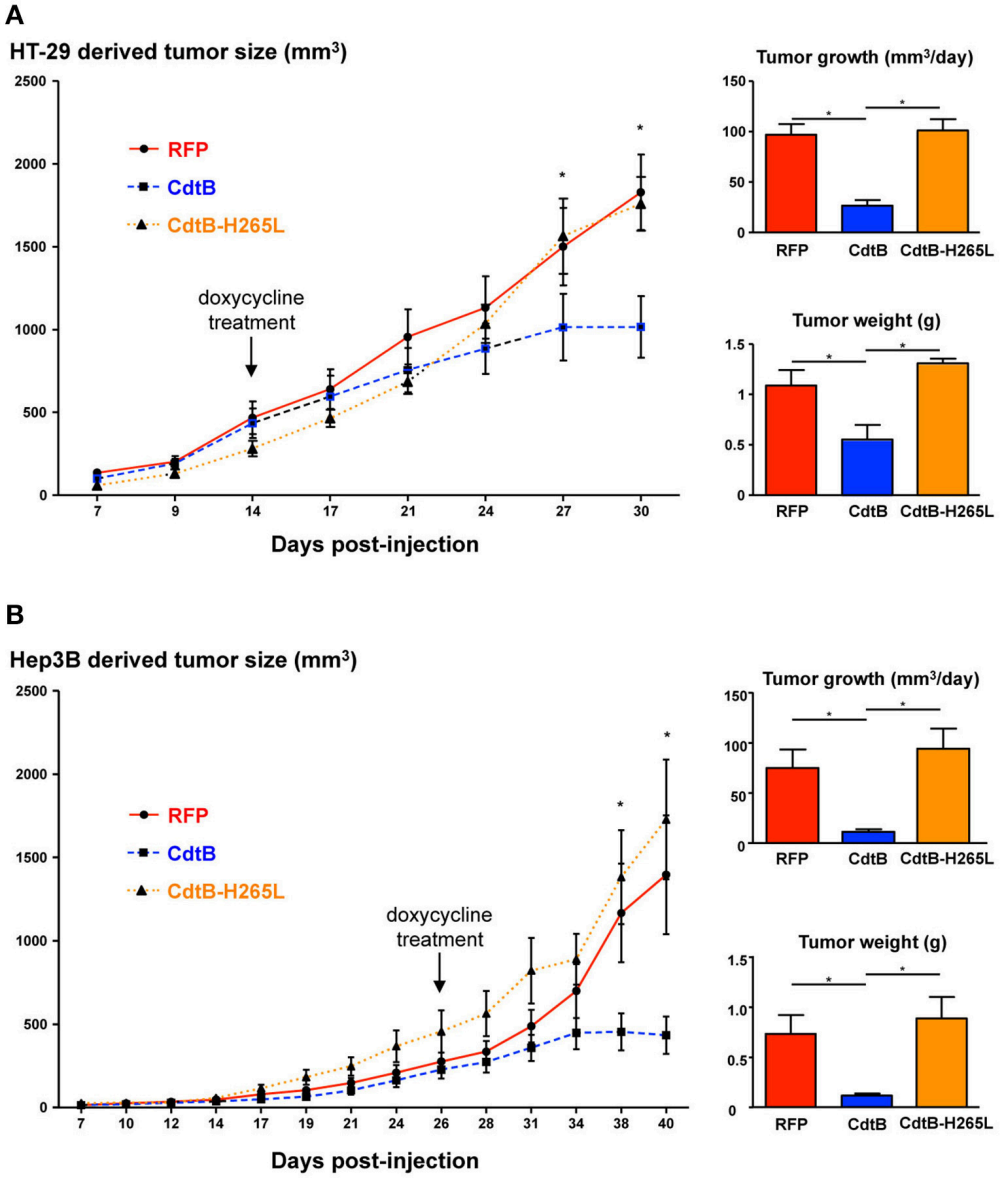
Effects of the CdtB subunit of *Helicobacter hepaticus* CDT on tumor growth. HT-29 (2 × 10^6^ cells) and Hep3B (4 × 10^6^ cells) were injected subcutaneously into the flank of immunodeficient NSG and tumor size was monitored 3 times per week. When the engrafted HT-29- and Hep3B-derived tumors reached approximately 400 and 200 mm^3^, respectively, doxycycline was administered *per os* permanently to all of the mice. **(A)** HT-29- and **(B)** Hep3B-derived tumor growth in mice. The curves represent the tumor growth. The daily tumor growth was also calculated from the beginning of the doxycycline treatment until the sacrifice of mice. The tumor weight was also measured at necropsy. The arrows indicate the first day of administration of doxycycline. CdtB, CdtB of *H. hepaticus* strain 3B1. CdtB-H265L, *H. hepaticus* CdtB with H265L mutation; RFP, red fluorescent protein. ^*^p < 0.05.

After successful engraftment of the transgenic cell lines, transgene expression was induced permanently with *per os* doxycycline during 2 weeks (indicated by arrows in Figure 2). Constant tumor growth was observed in control RFP mice while the tumor growth was significantly delayed in CdtB-mice for the last two tumor measurements corresponding to 6 days. The calculated daily tumor growth confirmed these results. Results for the CdtB-H265L-mice were similar to those measured for the control RFP-mice. At necropsy, the average tumor weight for CdtB-mice was at least 2–6 times less (HT-29 and Hep3B, respectively) than that of the RFP and CdtB-H265L mice for both tumor-derived cell lines. It should, however, be emphasized that the CdtB tumor size measured before necropsy was twice as big as that measured at the time of doxycycline induction.

### Analysis of engrafted tumors

**Figure 3:**
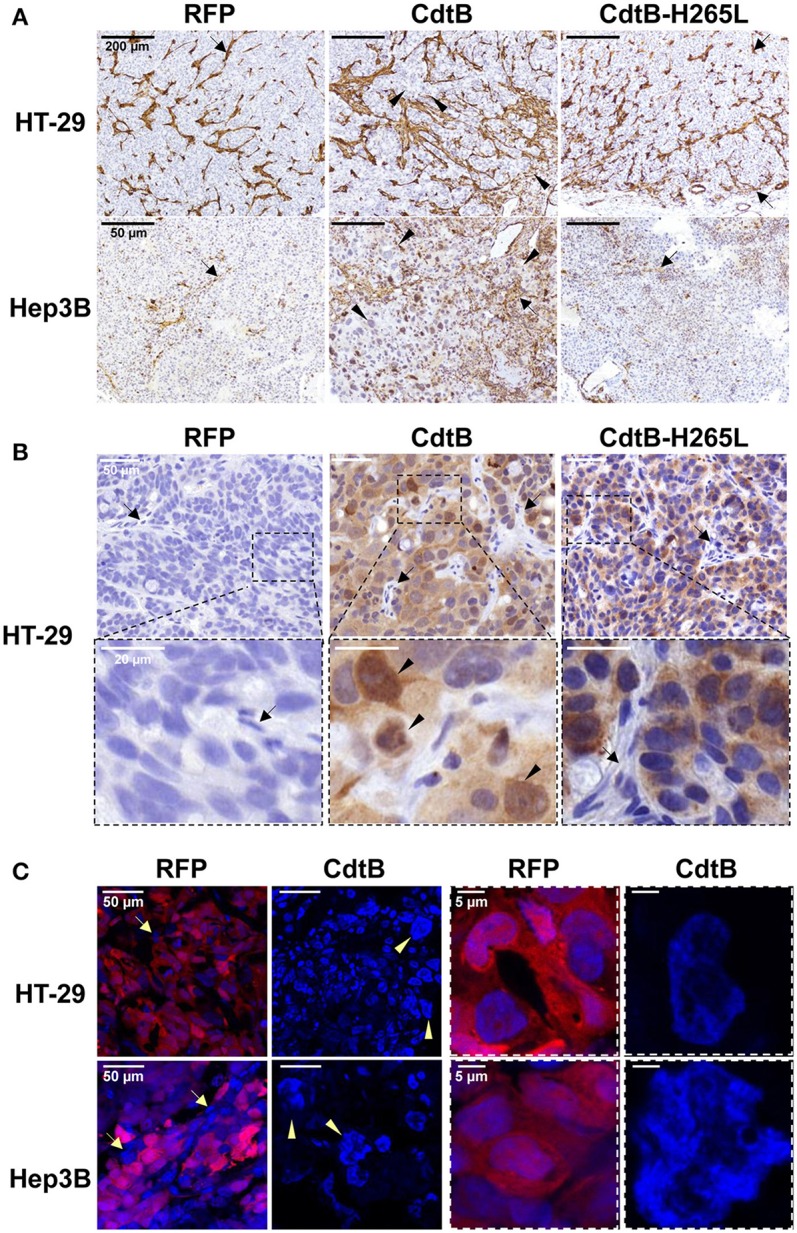
Detection and effects of *Helicobacter hepaticus* CdtB expression in tumor xenografts. Three μm-tissue sections of HT-29- and Hep3B-derived tumors were prepared from formalin-fixed paraffin-embedded tissues and submitted to standard hematoxylin staining and immunostaining raised against alpha smooth muscle actin **(A)** and HA **(B)** to detect murine infiltrates and the HA-tagged proteins (CdtB and CdtB H265L in HT-29-derived tumors), respectively. Boxes in **(B)** correspond to enlargement. **(C)** RFP fluorescence was visualized in 5 μm-tissue sections of HT-29- and Hep3B-derived tumors prepared from OCT-embedded tissues using a confocal microscope. Nuclei were counterstained with 4′,6-diamidino 2-phenylindole (DAPI) (blue). Boxes correspond to enlargement of cells and nuclei for RFP-derived tumors and to giant nuclei for CdtB-derived tumors. Tips of arrows indicate distended nuclei as compared to the RFP control or CdtB-H265L tumors. Arrows point to murine cell infiltrates. CdtB, CdtB of *H. hepaticus* strain 3B1; CdtB-H265L, *H. hepaticus* CdtB with H265L mutation; RFP, red fluorescent protein.

Overall, the CdtB-derived tumors were highly infiltrated by mice stromal cells compared to RFP- and CdtB-H265L-derived tumors (Figure 3A); these infiltrates appeared particularly important at the periphery of the HT-29-CdtB-tumors (**Figure 5D**). The *in vivo* expression of the respective transgenes was explored. Analysis of HT-29- and Hep3B-derived tumors using *cdtB*-based RT-qPCR revealed similar overall levels of *cdtB* and *cdtB*-H265L mRNAs, while higher levels of RFP mRNA were observed (Figure S1). Tumor histology analyses showed that the CdtB induced a cellular and nuclear enlargement in HT-29- and Hep3B-derived tumors (Figures 3A–C, tips of arrows). These effects were not observed for tumors expressing the RFP or the CdtB-H265L. Histological analysis revealed that overall the multi-HA tag was detected in both the cytosol and the nuclei of the cells from the HT-29-derived tumors, confirming the expression of the CdtB and CdtB-H265L, as previously reported in a lentiviral system of constitutive expression of these recombinant proteins (Péré-Védrenne et al., 2016). The most intense staining was observed in the most distended nuclei. In Hep3B, HA-labeling generated a significant background noise in the cytoplasm, preventing efficient analysis of Hep3B-derived tumors. Expression of RFP was visible to the naked eye in both tumor-derived RFP-expressing cell lines (not shown) reflecting the high stability of this protein (Merzlyak et al., 2007). Direct RFP fluorescence was detected by microscopy from tumors embedded in the OCT-compound. It revealed variable levels of red fluorescence, most likely reflecting the variable numbers of integrated RFP transgenes (Figure 3C). Similar cellular heterogeneity was observed regarding the CdtB expression in HT-29 derived tumors (HA labeling in Figure 3B). DAPI staining also showed a cellular and nuclear enlargement in response to the CdtB in contrast to tumor-derived RFP-expressing cells (Figure 3C), the shape of the giant nuclei being less rounded than that observed in non-intoxicated nuclei. Taken together, these data show that the transgenes were transcribed into mRNA and that the corresponding proteins (RFP, CdtB, and CdtB-H265L) were expressed *in vivo*.

**Figure 4:**
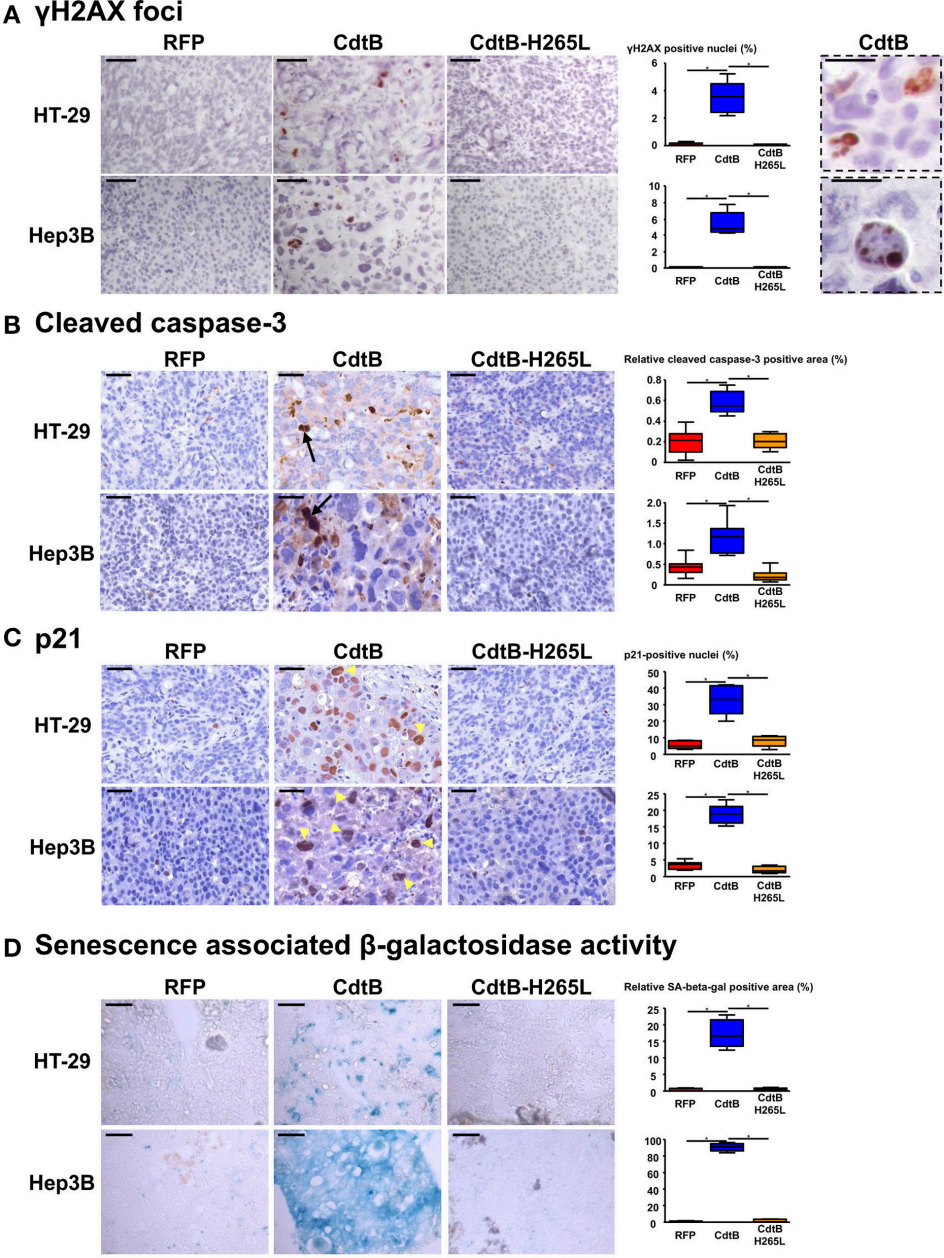
Effect of *Helicobacter hepaticus* CdtB expression in tumor xenografts. Three μm-tissue sections of HT-29- and Hep3B-derived tumors were prepared from formalin-fixed paraffin-embedded tissues and submitted to standard hematoxylin staining and immunostaining raised against γ-H2AX foci **(A)**, cleaved caspase-3 **(B)** and p21 **(C)**. Scale bars, 50 μm **(A–C)**. Subsequent counting of γ-H2AX- and p21-positive nuclei was performed with ImageJ 1.51 (Schindelin et al., 2015), each count being performed on 1,000 cells per mice. Relative cleaved caspase-3-positive area was quantified using the Mercator system image (Explora Nova, La Rochelle, France) for analysis of histological sections. Boxes in **(A)** correspond to enlargement of γ-H2AX-positive cells. Scale bar, 20 μm. Arrows and tips of arrows indicate cleaved caspase-3-positive cells and p21-positive distended nuclei, respectively. **(D)** Detection of the β-galactosidase activity in senescent cells. Five μm-tissue sections of HT-29- and Hep3B-derived tumors prepared from OCT-embedded tissues were submitted to β-galactosidase staining. The quantification of the β-galactosidase activity was determined with Image J 1.51 (Schindelin et al., 2015) by counting the positive area with respect to the total area. Scale bar, 100 μm; CdtB, CdtB of *H. hepaticus* strain 3B1; CdtB-H265L, *H. hepaticus* CdtB with H265L mutation; RFP, red fluorescent protein. ^*^p < 0.05.

An increase in γ-H2AX foci formation, a surrogate marker for double-stranded DNA breaks, was detected in CdtB-derived tumors (Figure 4A), confirming the DNA-damaging activity of CdtB in both cell lines. CdtB is a dual-function enzyme that can also act as a phosphatidylinositol-3,4,5-triphosphate (PIP3) phosphatase, perturbing PI-3K/Akt signaling (Shenker et al., 2014; Scuron et al., 2016). Phosphorylation of Akt (Ser473) and GSK-3β (Ser9) was evaluated with antibodies suitable for immunohistochemistry. For both HT-29 and Hep3B derived tumors, these staining revealed heterogeneity with stained and unstained cells and staining appeared to be more intense in intoxicated cells (Figures S2, S3). We also evaluated the mTOR downstream effector, S6 ribosomal protein (rpS6), since this protein is phosphorylated upon Akt activation and is considered as one of the most reliable readouts for PI3K activation (Ruggero and Sonenberg, 2005). Overall, rpS6 phosphorylation was observed mainly at the periphery of the tumors in both cell lines. In response to the CdtB, a significant increase of cells positives for rpS6 phosphorylation was observed in HT-29-tumors while this effect was less pronounced in Hep3B-derived tumors. Moreover, the staining reached deep into the center of the tumor for both cell lines, in accordance with the increased infiltration of stromal cells (Figure S4).

The expression of certain proteins involved in apoptosis, cell proliferation, cell cycle regulation, and senescence was analyzed by histology with subsequent quantification of positive cells. It was not possible to study the effect of the tumor suppressor triggering apoptosis p53. Indeed, the TP53 gene is mutated in HT-29 (Rodrigues et al., 1990) resulting in a high level of constitutive p53 protein expression (Figure S5A) while the TP53 gene was partially deleted in Hep3B (Bressac et al., 1990) and the corresponding protein p53 was therefore not detected with the antibodies used (Figure S5A). The detection of the large fragment of activated caspase-3 resulting from cleavage adjacent to Asp175 is indicative of apoptosis. A significant increase of cleaved caspase-3 was detected in all of the CdtB-tumors compared to the RFP and CdtB-H265L tumors (Figure 4B).

The cyclin-dependent kinase inhibitor 1A, p21 (p21Cip1 or p21Waf1), is a negative regulator of cell cycle progression (G1/S and G2/M transitions) mediating growth arrest, apoptosis and cellular senescence. p21 nuclear detection was increased almost 7-fold in response to the CdtB (Figure 4C). Interestingly, the most intense p21 staining was detected in the larger nuclei from giant cells (arrow tips indicated in Figure 4C). p21 can also directly interact with the proliferating cell nuclear antigen (PCNA), a DNA polymerase cofactor necessary for DNA replication in S phase and DNA damage repair, thus inhibiting cell proliferation. However, PCNA histology revealed an intense basal expression in the RFP control tumors for both cell lines which obstructed the visualization of the CdtB effects (Figure S5C). These data suggest that the CdtB is associated with an increase in p21-expressing and apoptotic cells and that the catalytic site of CdtB is involved in these effects.

Although apoptosis usually occurs in response to the CDT, for the *H. ducreyi* CDT (Blazkova et al., 2010; Kosar et al., 2011) some surviving cells exhibit cellular senescence markers, such as an increase in β-galactosidase activity and an overexpression of cell cycle inhibitors, such as p53, p21, and p16INK4a (the cyclin-dependent kinase inhibitor 2A regulating cell cycle progression and a biomarker of cellular senescence). As p16INK4a was expressed at high levels under all of the conditions (immunostaining in Figure S5B), it was not contributive and therefore the detection of β-galactosidase activity was performed to evaluate senescence. High β-galactosidase activity was induced in CdtB-tumors as compared to RFP- and CdtB-H265L-tumors, this effect being more important for Hep3B compared to HT-29 (Figure 4D). Taken together, these results demonstrate that the *H. hepaticus* CdtB induced a strong senescence in Hep3B and to a lesser extent in HT-29 cells.

**Figure 5:**
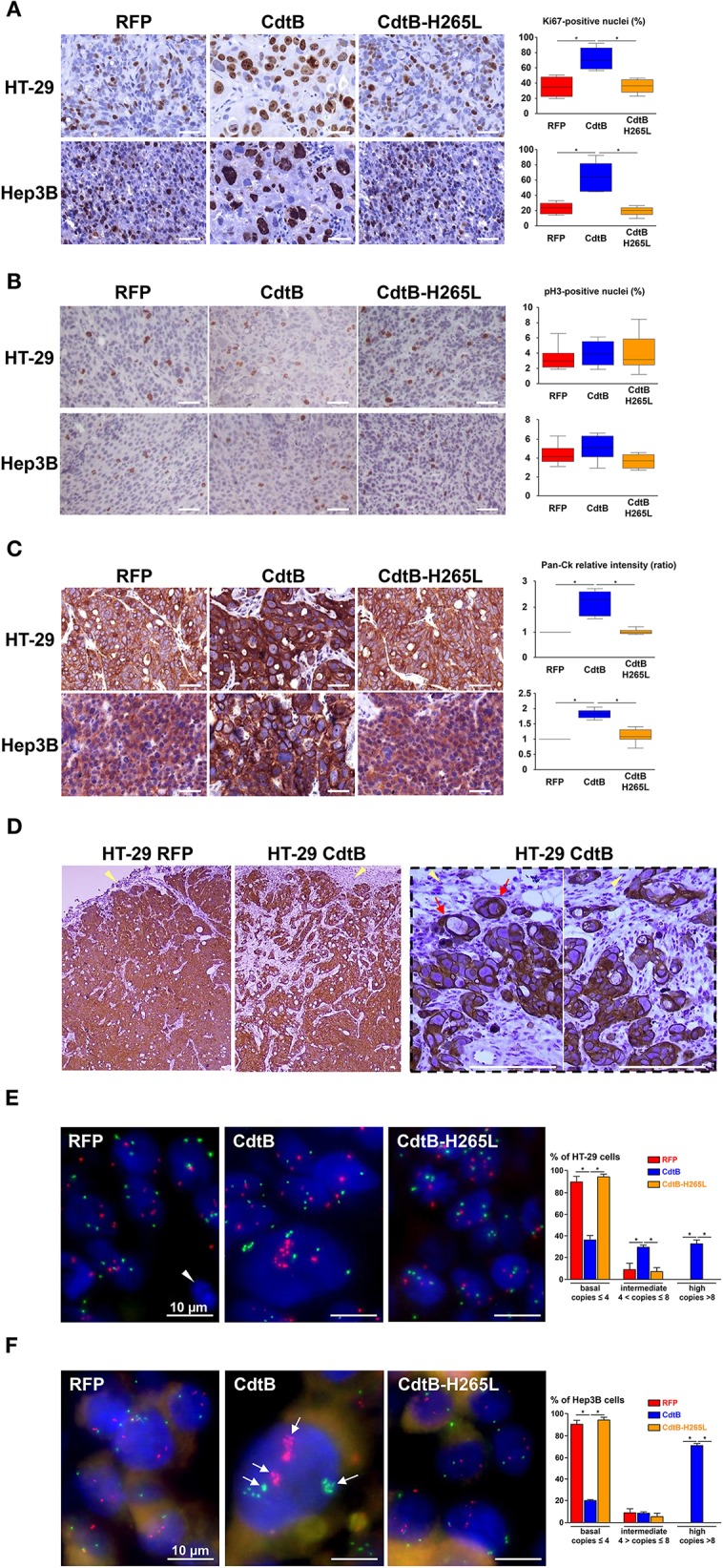
Effect of *Helicobacter hepaticus* CdtB expression in tumor xenografts. Three μm-tissue sections of HT-29- and Hep3B-derived tumors were prepared from formalin-fixed paraffin-embedded tissues and submitted to standard hematoxylin staining and immunostaining raised against primary anti-human Ki-67 **(A)**, phospho-histone H3 **(B)**, anti-human cytokeratin 5, 6, 8, 10, 13, and 18 (Pan-CK) **(C,D)**. Counting of Ki-67- and phospho-histone H3-positive nuclei, as well as quantification of cytokeratin intensity was performed with Image J 1.51 (Schindelin et al., 2015), each count being performed on 1,000 cells per mice. The results for the quantification of cytokeratins are presented as the ratio of the staining intensity normalized to RFP. Yellow tips of arrows in **(D)** indicate mice stromal cell infiltrates at the periphery of the HT-29-CdtB-tumors and the long red arrow points to individualized cell clusters at the periphery of HT-29-CdtB-derived tumor. Scale bars, 50 μm. FISH experiments (dual-color hybridizations) of 6 μm-tissue sections from formalin-fixed paraffin-embedded HT-29- **(E)** and Hep3B-tumors **(F)** were conducted with labeled probes hybridizing to the band on the short arm of human chromosome 5 (chromosome region 5p15.2, green) and to the chromosome 15 centromere (red). Each fluorescent spot corresponds to one copy of the chromosome region. Nuclei were counterstained with DAPI (blue). Mice nuclei are not labeled with these 2 human probes. White tips of arrows indicate non-labeled murine cells. In some Hep3B giant cells, foci containing a high number of chromosome copies are accumulated in different regions (long arrows). Scale bars, 10 μm. Three groups were determined for each condition, based on FISH signals counts: basal level (≤ 4 signals per probe per nuclei), intermediate (>4 and ≤8) and high level (>8). The basal level was in accordance to hypertriploid karyotypes of Hep3B and HT-29 cell lines. CdtB, CdtB of *H. hepaticus* strain 3B1; CdtB-H265L, *H. hepaticus* CdtB with H265L mutation; DAPI, 4′,6′-diamidino-2-phenylindol; RFP, red fluorescent protein. ^*^p < 0.05.

The nuclear antigen KI-67, a cell proliferation marker present during all active phases of the cell cycle (G1, S, G2, and mitosis), was also detected and quantified in all of the tumor groups (Figure 5A). The number of Ki-67-positive nuclei was increased 2-fold in response to the CdtB vs. RFP and CdtB-H265L (Figure 5A). In the CdtB tumors, Ki-67 was mostly detected in enlarged nuclei; more nuclei were bigger and Ki-67 labeling was more intense. This increase in Ki-67-positive cells most likely reflects the expression of Ki-67 accumulated in the G2 phase following the CdtB-induced G2/M cell cycle arrest. Indeed, non-cycling cells arrested in G2/M were previously reported to be Ki-67-positive (Lundblad et al., 1991; van Oijen et al., 1998). Thus, a more specific marker of proliferating cells undergoing mitosis, phospho-histone H3, was evaluated. It revealed a moderate labeling and no significant changes were observed between the different conditions (Figure 5B). It should be nevertheless be emphasized that some enlarged cells were positive for phospho-histone H3 labeling in the CdtB-derived tumors, suggesting that some CdtB-intoxicated cells undergo mitosis (Figure 5B).

Markers of epithelial cell differentiation were analyzed (Figure 5C). Cytokeratins are intermediate filament proteins in the intracytoplasmic cytoskeleton of epithelial cells. Human cytokeratins 4, 5, 6, 8, 10, 13, and 18 were simultaneously detected using anti-cytokeratin pan reactive antibody. Regarding Hep3B, cytokeratins appeared to be expressed homogeneously in the cytoplasm of RFP- and CdtB-H265L-derived tumors while a more intense labeling was detected around the nuclei of CdtB-derived tumors. In HT-29-derived tumors, cytokeratins were concentrated in perinuclear regions and in the cell-cell junctions and a more intense labeling was observed in response to the CdtB. It was not possible to quantify these cytokeratins in western blot experiments because the CdtB-derived tumors were highly infiltrated with mice stromal cells compared to RFP- and CdtB-H265L-derived tumors (Figures 3A, 5D), hampering human protein quantification required for western blot normalization. In addition, some cell clusters, strongly expressing cytokeratins and apparently detached from the tumor, were detected at the periphery of only HT-29-CdtB-derived tumors (Figure 5D). These cell clusters were localized at the invasion front of the tumors spreading into the surrounding stroma and suggest that these enlarged CdtB-intoxicated cells remain nevertheless aggressive or/and invasive.

As polyploidy has been proposed to protect against DNA damage (Zheng et al., 2012; Fox and Duronio, 2013), cellular chromosome content was evaluated in DNA FISH experiments using 2 probes targeting two different chromosomes. Overall, we observed significant differences in FISH signal counts between CdtB vs. RFP and CdtB-H265L conditions for both Hep3B and HT-29 engrafted tumors. An increase in chromosome number was detected mainly in enlarged nuclei in both CdtB-derived tumors (Figures 5E,F). The largest nuclei were mostly located at the periphery of the CdtB-tumors. In HT-29, an average of 2–4 chromosome spots per nucleus was observed for RFP- and CdtB-H265L-derived tumors while the spot number almost doubled in response to the CdtB in more than 30% of the cells. In Hep3B, 2–4 chromosome spots were counted per RFP- and CdtB-H265L-nucleus while a 10-fold increase in spot number was observed in response to CdtB. In more than 70% of the Hep3B giant cells, foci containing a high number of chromosome copies accumulated in different regions, reflecting the absence of chromosomes segregation. Giant cells were observed for Hep3B-derived tumors but smaller cells with an intermediary degree of ploidy were also present in those tumors (Figure 5F).

## Discussion

In the present study, the direct effects of the active CdtB subunit of the CDT were evaluated using colon and liver epithelial cell lines allowing the inducible expression of the *H. hepaticus* CdtB. *In vitro*, this lentiviral model reproduced the well-known effects of the *H. hepaticus* CdtB, i.e., a major remodeling of the cytoskeleton as well as the nuclear translocation of NF-κB (Young et al., 2000; Péré-Védrenne et al., 2016). The transduced cells were then engrafted into immunodeficient mice and the CdtB subunit was conditionally expressed. In this xenograft model, the CdtB expression was associated with a delayed tumor growth, confirmed by the tumor weight at necropsy. In contrast to what was observed *in vitro*, some CdtB-intoxicated cells survived until the sacrifice of the mice, 2 weeks later. In addition, the size of the tumors continued to increase (nearly 2-fold) during this period, suggesting the bypass of a complete cell cycle arrest.

In the present study, CdtB expression was also associated to an increase of γH2AX-foci for both engrafted cell lines, attesting double-stranded DNA breaks, as reported in infected-mice in which CDT also triggers γ-H2AX foci formation (Ge et al., 2017). Other studies reported that *in vitro* CDT intoxication modulates Akt signaling within a few hours of exposure. *A. actinomycetemcomitans* CDT rapidly decreases phospho-Akt and phospho-GSK-3β in infected macrophages (Shenker et al., 2014) while *C. jejuni* CDT would not be a critical factor for the activation of PI3K/Akt pathway during infection (Li et al., 2011). For both engrafted cell lines, phosphorylation of Akt (Ser473) and GSK-3β (Ser9) appeared to be increased in response to the CdtB that would be in agreement with Akt activation in the DNA damage response pathway (Liu et al., 2014). Phospho-rpS6, the critical downstream effector of mTOR, another downstream effector of PI3K, was also increased in response to CdtB. Phosphorylation of rpS6 is induced by many stimuli and is involved in the control of cell size and cell-cycle progression, as well as in translational control of particular classes of mRNAs (Ruggero and Sonenberg, 2005; Ruvinsky and Meyuhas, 2006). Dephosphorylation of rpS6 occurs during growth arrest while its phosphorylation is associated with cell size increase (Fingar et al., 2002). Phosphorylation of rpS6 is also associated with DNA damage and DNA repair signaling (Sun et al., 2014). Therefore, the longer-term AKT, GSK-3β, and rpS6 phosphorylation observed in the CdtB-expressing tumors likely reflects the DNA damage response to the CdtB. It is thus possible that some CDT are more dependent upon DNase activity to induce toxicity, as previously proposed by Scuron et al. (2016).

CdtB expression was associated with an increase in apoptosis, senescence and p21 expression. *In vitro*, it was shown that primary cells, fibroblasts, endothelial cells, hematopoietic and epithelial cells exposed to CDT undergo apoptosis after the acute phase of CDT intoxication (Wising et al., 2005; Smith and Bayles, 2006). However, the CDT-induced apoptosis depends on the cell type, in particular the p53 status, the toxin dose and the incubation time. Indeed, the CDT induces *in vitro* apoptosis and necrosis in 90% of the infected T cells and monocytes, while apoptosis ranged from 26 to 32% in epithelial cells, keratinocytes and fibroblasts, as demonstrated for the *H. ducreyi* CDT (Wising et al., 2005). In a mouse xenograft model using a prostate cancer cell line, a high-dose of CDT was shown to inhibit cell growth and to induce apoptosis (Lai et al., 2014). The apoptosis induced by the CDT toxin is mainly mediated by the ATM pathway, in a p53 activation dependent or independent manner (reviewed in Guerra et al., 2011). The CDT-induced apoptosis can also be mediated via a mitochondria-dependent activation of the caspase cascade (Ohara et al., 2008; Liyanage et al., 2010). In the present tumor xenograft model, no CdtB-p53 regulation was noted since the p53 gene is mutated in both HT-29 and Hep3B cell lines, suggesting that the observed CdtB-induced apoptosis was p53-independent. It should however be noted that *in vitro* assays performed with the non-mutated p53 hepatic HepG2 cell line revealed similar CdtB effects (G2/M cell cycle arrest, NF-κB nuclear translocation, distended nuclei, Figures S6A–C) as those observed for HT-29 and Hep3B cell lines. In HepG2, no significant regulation of cleaved caspase 3 was observed while p53 was expressed into the distended nuclei (Figure S6D), suggesting that the CdtB effects were at least in part dependent on p53 signaling.

Apoptosis does not affect all of the CDT-intoxicated cells since normal and cancer-derived cells can survive the acute phase of intoxication and enter cellular senescence, as demonstrated by the *H. ducreyi* CDT *in vitro* (Blazkova et al., 2010; Kosar et al., 2011). Senescence is characterized as a program of restricted proliferative capacity, which is manifested by broad morphological and biochemical changes, as well as an irreversible cell cycle arrest. In the present xenograft models, senescence undoubtedly occurred very strongly, as demonstrated by the increase in β-galactosidase activity and the increased expression of the nuclear cell cycle inhibitor p21. The secreted chemokines and pro-inflammatory cytokines usually associated with senescent cells could not be tested in this xenograft model since mice were immunodeficient and the expression of the transgenes was induced with doxycycline, an anti-inflammatory agent (Berman et al., 2007). However, we previously reported that CdtB triggers IL1β, IL6, IL8 in HT-29, and Hep3B cells *in vitro* (Péré-Védrenne et al., 2016), reinforcing the putative role of the CdtB-associated secretome in the remodeling of the surrounding stroma.

Cells that enter mitosis after DNA damage frequently undergo aberrant mitosis (mitotic catastrophe), which may lead either to cell death through apoptosis or necrosis or to senescence, apparently as a result of stabilization of the G1 tetraploidy checkpoint. Cancer cells that undergo *in vitro* senescence become giant, polyploid and cease to proliferate (reviewed in Shay and Roninson, 2004). Increased polyploidy in response to the CdtB could thus occur from senescent cells. If senescent cancer cells are often polyploid, their route to polyploidy is poorly recognized (endoreplication vs. aberrant mitosis).

The increased expression of the cell cycle proliferation marker Ki-67 as well as the cyclin-dependent kinase inhibitor 1, p21, in the bigger cells suggests that the CdtB triggers p53-independent cell endoreplication from p21-activated G2 arrest leading to giant cells (Bates et al., 1998). In endoreplication cell cycles, cells with replicated genomes bypass mitosis and successively replicate their genomes without segregating chromosomes during mitosis and thereby become big polyploid cells. The high increase of ploidy in the giant cells supports this hypothesis. If endoreplication plays a role in modulating stress cell response, the transition from mitosis to endoreplication is also involved in cell differentiation and morphogenesis in developing tissues (see Orr-Weaver, 2015 and references cited therein) and data suggest that endoreplication and polyploidy are required for the maintenance of cell identity. The intense labeling of the epithelial cell differentiation markers, cytokeratins, observed in CdtB-derived tumors and endoreplication could also be triggered to maintain CdtB-intoxicated cell identity in response to major actin cytoskeleton remodeling. Thus, endoreplication induced in response to the CdtB could be an adaptation to genotoxic stress as well as a mechanism induced for differentiation of polyploid cells, as recently proposed (Fox and Duronio, 2013).

Since non-cycling cells arrested in G2/M are known to be Ki-67-positive (Lundblad et al., 1991; van Oijen et al., 1998), we assumed that the increase in Ki-67-positive nuclei observed in CdtB-tumors likely reflects the accumulation of Ki-67 in non-proliferating cells arrested in G2/M. In the present study, all of the cells are believed to express the CdtB at different doses as observed for RFP. Indeed, puromycin selection was maintained *in vitro* for more than 60 days prior to engraftments and histological analyses revealed that the multi-HA tag was detected in all of the cells of the HT-29-derived tumors. Some of these Ki-67-positive cells could also correspond to cells with a low *cdtB* transgene copy in which (1) CdtB could exhibit a poor DNase activity and would not be active enough to overcome the DNA repair machinery of those cells or (2) DNA repair mechanisms were efficient. The presence of cells that underwent mitosis in those xenograft models supports this hypothesis. Indeed, it was recently demonstrated *in vitro* that cells exposed to sub-lethal doses of *H. hepaticus* or *H. ducreyi* CDT present on one hand genomic instability and altered DNA damage response and on the other hand normal cell cycle distribution with no decrease in viability nor phenomenon of senescence (Guidi et al., 2013), suggesting that chronic CDT intoxication promotes the survival and proliferation of cells carrying impaired mechanisms of detection and repair of DNA damage (Grasso and Frisan, 2015). The increase in the size of engrafted tumors after 2 weeks of CdtB induction, although very attenuated compared to RFP- and CdtB-H265L-tumors, nevertheless supports this hypothesis.

In these newly implemented and original *in vivo* xenograft mouse models, the expression of the *H. hepaticus* CdtB induces some of the different CDT effects observed *in vitro*, such as γ-H2AX foci formation, p21 regulation, apoptosis and senescence. In addition, CdtB triggers large murine stromal cell infiltrates and clusters of cells are detached from the invasion front of the tumors into the surrounding stroma, a feature of aggressiveness in cancer. An increased expression of several cytokeratins (epithelial cell differentiation markers) as well as an increase in ploidy were also observed. Our data suggests that the CdtB promotes senescence and cell endoreplication leading to giant polyploid cells. Endoreplication is also known to confer genome instability, a major cancer-enabling property (Fox and Duronio, 2013). The exact involvement of endoreplication induced in response to the toxin in the cancer “activation-protection” balance remains to be determined.

In conclusion, we demonstrated that cancer cells can survive and adapt to prolonged exposure to CdtB. This resistance involves cellular senescence and polyploidization. Therefore, the potential role of CdtB in tumor initiation and/or tumor progression deserves to be studied in greater detail.

## Author contributions

CPV performed the vector construction and established the transgenic cell lines. She was involved in all the steps of this project. MPC performed DNA FISH experiments and fluorescence analysis of tumors embedded in the OCT-compound. BR injected the cells into the mice and monitored tumor growth 3 times per week by measuring tumor size. WH established conditional transgenic HepG2 cell line and performed *in vitro* experiments with HepG2. LC and ES performed tumor histology analyses. AB performed RT-qPCR experiments. PD is a pathologist and helped to write the manuscript. FM is the director of the laboratory. He participated in the design of the study. CV is an expert in tumor histology and was involved in tumor analysis and the writing of the manuscript. AM is the group leader and the principal investigator of the project and the PhD supervisor of CPV.

### Conflict of interest statement

The authors declare that the research was conducted in the absence of any commercial or financial relationships that could be construed as a potential conflict of interest.
