# Supplementary material for: The Cytolethal Distending Toxin Subunit CdtB of Helicobacter hepaticus Promotes Senescence and Endoreplication in Xenograft Mouse Models of Hepatic and Intestinal Cell Lines
Source: Front Cell Infect Microbiol. 2017 Jun 30;7:268. doi: 10.3389/fcimb.2017.00268 (PMC5491915; doi:10.3389/fcimb.2017.00268)
Supplement: Supplementary file 1 [file DataSheet1.pdf]

SUPPLEMENTARY MATERIALS & METHODS

Cell lines and culture, Helicobacter hepaticus strain and culture. Cell lines were maintained in culture medium supplemented with 10% heat-inactivated fetal calf serum (FCS, Invitrogen, Cergy Pontoise, France) at 37°C in a 5% CO_2 humidified atmosphere. HT-29 human colorectal adenocarcinoma cells (DSMZ no. ACC 299, DSMZ collection) were cultured in McCoy's 5a medium (Invitrogen, Life Technologies, Courtaboeuf, France). Hep3B human hepatocellular carcinoma cells (ATCC® HB-8064™) were grown in Dulbecco's Modified Eagle's medium (Invitrogen).

H. hepaticus strain 3B1 (3B1/Hh-1, CIP 104102, ATCC 51449, CCUG 44777) was cultured as previously reported (Le Roux-Goglin et al. 2013). DNA was extracted using the QIAamp DNA Mini Kit (Qiagen SAS, Courtaboeuf, France).

Reagents and antibodies. Puromycin and doxycycline were purchased from Invitrogen and Sigma Aldrich, respectively. Monoclonal mouse anti-human Ki-67 antigen (clone MIB-1) and monoclonal mouse anti-human p53 protein (clone DO-7) were provided from DAKO (Les Ulis, France). Monoclonal rabbit anti-cleaved caspase-3 (Asp175) (clone 5A1E), polyclonal rabbit anti-phospho-histone H3 (Ser10), monoclonal rabbit anti-phospho-histone H2A.X (Ser139) (clone 20E3), monoclonal rabbit anti-phospho-Akt (Ser473) (clone D9E, antibody #4060) and polyclonal rabbit anti-phospho-S6 (Ser235/236) (antibody #2211) were purchased from Cell Signaling (Danvers, MA, US). Monoclonal mouse anti-human cytokeratin (pan reactive) (clone C11), polyclonal rabbit anti-human NF-κB p65 (clone C20), monoclonal mouse anti-HA (clone 16B12) and monoclonal mouse anti-human p21 (clone 6B6) were provided from BioLegend (London, UK), Santa Cruz Biotechnology (Santa Cruz, CA, USA), Covance (Emeryville, CA, US) and BD Pharmingen™/BD Biosciences (Le Pont de Claix, France), respectively. Monoclonal mouse anti-CDKN2A/p16INK4a (clone 2D9A12, ab54210), polyclonal rabbit anti-phospho-GSK-3β (Ser9) (clone EPR2286Y, ab75814) and

monoclonal mouse anti-alpha smooth muscle actin (clone 1A4, ab7817) antibodies were purchased from Abcam (Paris, France). Alexa Fluor 488-labeled secondary antibodies, Alexa Fluor 647-labeled phalloidin, and 4',6'-diamidino-2-phenylindol (DAPI) used for immunofluorescence were purchased from Molecular Probes (Eugene, OR, USA). ImmPRESS™ HRP anti-mouse IgG (peroxidase) polymer detection kit (Vector Laboratories, Laboratoires Eurobio/Abcys, Les Ulis, France) and EnVision+ System- HRP Labelled Polymer anti-rabbit (DAKO) were used as secondary antibodies for histology experiments.

Construction of the plasmids used for the lentivirus production. The *cdtB* sequence and the corresponding mutated *cdtB* sequence (A→T transversion at nucleotide 794 (Avenaud et al. 2004) corresponding to the His→Leu mutation at residue 265 involved in the catalytic activity) of *H. hepaticus* strain 3B1 fused at their 3' end to three repeats of the human influenza hemagglutinin (HA) epitope (*cdtB*-3HA, GenBank accession numbers: KT590046 and KT590047, respectively) were cloned into the pTRIPz lentiviral plasmid (supplied by the Structure Fédérative de Recherche TransBioMed, University of Bordeaux, France) instead of the Turbo Red Fluorescent Protein (RFP) gene and the shRNAmir cloning site. The *cdtB*-3HA sequences were amplified from p252 lentiviral plasmids, previously described (Péré-Védrenne et al. 2016), by PCR using primers (5'-GCTAGCGTTTAAACATCGATACCGGTCCCGGG**ATG**AGAATACTATTATGCTTTTTA-3' and 5'-TTAATTAATCGATGAATTCACGCGTACTAGT**TTA**AGCGTAATCTGGAACG-3') containing the restriction sites *Age*I and *Mlu*I (underlined in the primers) present in the pTRIPz plasmid. The *cdtB*-3HA-990-bp PCR product was first subcloned into the pGEM®-T Easy Vector System I (Promega, Charbonnières-les-Bains, France). After sequence verification, plasmids from positive clones were extracted using a QIAprep Spin Miniprep kit (Qiagen SAS). The pGEM-T-*cdtB,* pGEM-T-*cdtB* and the pTRIPz plasmids were digested successively using *Age*I and *Mlu*I restriction endonucleases (New England Biolabs, Evry, France). After migration on a 1% agarose gel, the DNA fragments of interest were recovered using a NucleoSpin Gel and PCR Clean-up kit (MACHEREY-NAGEL, Hoerdt, France). The

ligation of the *cdtB*-3HA sequence into the pTRIPz was conducted overnight at 4°C using a molar ratio insert/vector of 3 with 400 U of T4 DNA ligase (Promega) in a final volume of 10 µl. Transformation of the *Escherichia coli* ElectroMAX™ Stbl4™ Competent Cells (Invitrogen) was achieved with 1 µl of the ligation mixture, according to the manufacturer's recommendations. After a maximum of 16 h of incubation on agarose plates supplemented with 100 µg/ml ampicillin at 37°C, the plasmids were extracted using the QIAprep Spin Miniprep kit and digested by several restriction endonucleases (*Eco*RI, *Not*I, *Pvu*II, *Xma*I and *Ssp*I) to verify the absence of recombination events frequently observed when using unstable vectors. The sequence was finally verified using external primers designed from the pTRIPz (5'-GAGAACGTATGTCGAGGTAGGC-3') and 5'-GGGCGGAAGGATCAGGAC-3') and with internal primers designed on the *H. hepaticus cdtB* gene (5'-AGCAAGAAGGGTGAATCTCG-3' and 5'-TCATTGCCGATACGAATACC-3'). The plasmids were stored at -20°C. As the transformed cells cannot be preserved, new transformations of the ElectroMAX™ Stbl4™ Competent were performed prior to each maxi-preparation of plasmid with the NucleoBond Xtra Maxi EF kit (MACHEREY-NAGEL) with the verifications previously described (digestions and sequencing).

Production of lentivirus particles. The pTRIPz, pTRIPz-*Hh*-CdtB and pTRIPz-*Hh*-CdtB-H265 lentiviral vectors were used for stable cellular integration of the RFP, *H. hepaticus cdtB*-3HA*,* and *H. hepaticus cdtB*-3HA sequences. Lentiviral particles were produced from these plasmids by the service platform for lentiviral vector production of the Structure Fédérative de Recherche TransBioMed at the University of Bordeaux, as previously described (Varon et al. 2014). Viral titers of lentiviruses were determined by measuring the p24 antigen level (Varon et al. 2014). Approval was obtained from the French Committee of Genetic Engineering (approval number 4608).

Cell line establishment. Cells were seeded in 6-well plates at a density of $1x10^5$/well and incubated for 24 h. The culture medium was then removed and volumes corresponding to a

multiplicity of infection (MOI) of 30 viruses/cell in FCS-free renewed medium were added to each cell culture well for 12 h. Then FCS was added to a final concentration of 10% for another 12 h. The medium was then completely renewed with fresh medium with FCS and incubation was continued for 3 days. The cells were seeded in T25 tissue culture flasks. After adherence, the cells were permanently cultured in the presence of puromycin (2 µg/ml) to specifically select cells having integrated the sequences of interest and to establish a stable cell line carrying the transgenic sequence in a transcriptionally silent form.

Quantification of the number of integrated proviral copies in the cell lines was verified by quantitative PCR targeting the uroporphyrinogen III synthase (UROS) gene present in all constructs (Robert-Richard et al. 2010).

Immunofluorescence and image analysis. Cell cultures were grown on glass coverslips. When needed, coverslips were mounted on microscope slides with Fluoromount-G (Clinisciences SA, Montrouge, France) and treated as previously reported (Varon et al. 2009) with minor modifications. NF-κB nuclear translocation was analyzed by immunostaining with polyclonal antibodies generated against NF-κB p65 (dilution 1/100). Triple-color imaging with DAPI, phalloidin and Alexa 488-labeled secondary antibodies was obtained using selective laser excitation at 405, 647 and 488 nm, respectively. Traditional widefield fluorescence imaging and confocal microscopy were performed as previously reported (Péré-Védrenne et al. 2016).

Tumor processing. After mice sacrifice, tumors were immediately harvested and processed for further analyses; they were divided into 3 pieces. The first part of the tumor was directly snap frozen using liquid nitrogen and stored at -80°C. The second part was fixed immediately in 4% paraformaldehyde in phosphate buffered saline (PBS) (Invitrogen) for 4 h at room temperature, washed three times with PBS at room temperature and further incubated in 30% sucrose (Sigma Aldrich, Saint-Quentin Fallavier, France) in PBS overnight at 4°C; then the tumor was stored at -80°C in Tissue-Tek® OCT-compound (Sakura,

Labonord, Templemars, France). The third part of the tumor was fixed immediately in 4% formaldehyde in PBS for 24 h at room temperature and then washed three times with PBS; the tissues were subsequently submitted to standard histological processing and embedded in paraffin.

Tissue analysis, histology and immunohistochemistry. Three µm thick tissue sections were prepared from formalin-fixed paraffin-embedded tissues and submitted to standard hematoxylin staining (Sigma Aldrich) and immunohistochemistry protocols. Primary antibodies were incubated for 30 min (anti-Ki-67 and phospho-histone H3, dilution: 1/75 and 1/200), 1 h (anti-p16INK4a, p21, p53 and alpha smooth muscle actin; dilution: 1/500, 1/50 1/200 and 1/50, respectively) or 2 h (anti-cleaved caspase-3, cytokeratin (pan reactive), phospho-histone H2AX, HA, anti-phospho-Akt, anti-phospho-GSK-3β, anti-phospho-S6 dilution: 1/100, 1/100, 1/100, 1/200 and 1/300, respectively) at room temperature, followed by a 30 min incubation at room temperature with anti-mouse labeled polymer-HRP DAKO Envision Systems (DAKO). Immunolabeling was revealed after a 10 min incubation in liquid substrate-diaminobenzidine-chromogen (DAKO). Slides were counterstained with hematoxylin, dehydrated and mounted with Eukitt-mounting medium (Sigma). Histology was examined by an expert pathologist (P. Dubus). After analysis, the slides were scanned in their entirety using a digital slide scanner (Pannoramic SCAN; 3DHISTECH Ltd, Budapest, Hungary) for subsequent analysis. The images were read using the Pannoramic Viewer software version 1.15.4 (3DHISTECH Ltd). Quantification of histological sections was performed using the Mercator image analysis system (Explora Nova, La Rochelle, France) and ImageJ 1.51 (NIH). Images presented in the figures were recorded on tissue sections using light microscopy and a 4X to 20X objective.

Five µm thick tissue sections were prepared from fixed frozen optimal cutting temperature compound (OCT)-embedded tissues and submitted to β-galactosidase staining with the Senescence β-Galactosidase Staining kit (Cell Signaling), according to the supplier's

recommendations. Tissue sections were also directly examined using light microscopy to visualize the red fluorescence of the RFP.

Primer designs. PCR primers were designed using the software "Primer Express 2.0" (Applied Biosystems, Carlsbad, CA, USA) and synthesized by Eurofins MWG Operon (Courtaboeuf, France). The PCR primers used for the reference gene (HPRT1, accession number NM_000194.2) are F-HPRT1: 5’-TGGTCAGGCAGTATAATCCA-3’ and R-HPRT1: 5’-GGTCCTTTTCACCAGCAAGCT-3’. The PCR primers used for the RFP gene are F1-RFP: 5’-TCAAGGAGGCCGACAAAGAG-3’ and R1-RFP: 5’-GTACTTGGCCACAGCCATCTC-3’; those used for the *cdtB* gene of *H. hepaticus* are F2-Hh-cdtB: 5’-CAAGAGGCTGGCGCTATACC-3’ and R2-Hh-cdtB: 5’-CACCGGGCTGAACCATTC-3’.

Real Time-PCR experiments and quantification of the expression of RFP and *cdtB* genes. Total RNA samples were extracted from frozen tumors with the miRNeasy Mini Kit (Qiagen). The quality and concentration of the purified RNA was verified by optical density. The absence of residual DNA in the samples was verified by real time (RT)-PCR targeting the glyceraldehyde 3-phosphate dehydrogenase gene (GAPDH) under the PCR conditions described below with the forward (5’-GAAGGTGAAGGTCGGAGTC-3’) and reverse primers (5’-GAAGATGGTGATGGGATTTC-3’). RNA extracts were maintained at -80°C until use.

Reverse transcription of total RNA up to 2 µg into cDNA was achieved using the High Capacity cDNA Reverse Transcription kit (Applied Biosystems) following the manufacturer's recommendations. RT-PCRs for the target and reference (hypoxanthine phosphoribosyltransferase 1, HPRT1) cDNA were performed simultaneously under the conditions described below.

Each cDNA was analyzed in triplicate by RT-PCR in a 96-well plate using the LightCycler 480 (Roche Diagnostics, Meylan, France). For each PCR, 18 µl of PCR mixture containing 10 ng cDNA per PCR reaction and 12.5 µl of SYBR Green PCR Master Mix (Applied Biosystems) were used per well. Then, 2 µl of forward and reverse primers were added in

different wells to a final concentration of 1 µM. cDNA was amplified using the following cycling parameters: heating at 95°C for 10 min followed by 40 cycles of a three-stage temperature profile of 95°C for 10 s, 60°C for 15 s and 72°C for 30 s. At the end of the PCR, a melting step was performed to verify the presence of a single peak at the expected melting temperature confirming the specificity of the PCR and the absence of primer dimers. The option "Absolute Quantification" was chosen for the calculation of threshold cycles (Ct). For each gene, the PCR efficiency (E) was checked using dilutions of the sample to obtain a standard straight line and calculated from the slope of the standard straight line (p) with the following equation:

$E = 10^{-1/p}$.

The relative quantification of expression of the target gene was determined according to that of the HPRT1 gene used as a reference. The Ct values obtained were used to calculate a ratio with the $2^{-\Delta\Delta Ct}$ method (Livak et al. 2001). For the relative quantification of the target gene, the ratio $2^{-\Delta\Delta CT}$ was calculated from the following equation and was standardized with the HPRT1 gene:

$\Delta\Delta Ct = Ct\ (\text{target gene} - \text{reference gene})_{\text{reference cells}} - Ct\ (\text{target gene} - \text{reference gene})_{\text{target cells}}$

Statistical analysis. Statistical analyses were performed using GraphPad Prism version 5 (GraphPad software, San Diego, CA, USA). The results are presented as the mean ± standard deviation. The means were compared using a non-parametric test; the Mann-Whitney test, for comparison between two groups. A difference was considered significant when p was less than 0.05.

REFERENCES for datasheet

Avenaud P., Castroviejo M., Claret S., Rosenbaum J., Megraud F.Menard A. (2004) Expression and activity of the cytolethal distending toxin of *Helicobacter hepaticus*. Biochem Biophys Res Commun. 318, 739-745.

Le Roux-Goglin E., Dubus P., Asencio C., Jutand M. A., Rosenbaum J.Megraud F. (2013) Hepatic lesions observed in hepatitis C virus transgenic mice infected by *Helicobacter hepaticus*. Helicobacter. 18, 33-40.

Livak KJ, Schmittgen TD. (2001) Analysis of relative gene expression data using real-time quantitative PCR and the 2(-Delta Delta C(T)) Method. Methods. 25:402-8.

Péré-Védrenne C., Cardinaud B., Varon C., Mocan I., Buissonniere A., Izotte J., et al. (2016) The Cytolethal Distending Toxin Subunit CdtB of Helicobacter Induces a Th17-related and Antimicrobial Signature in Intestinal and Hepatic Cells In Vitro. J Infect Dis. 213, 1979-1989.

Robert-Richard E., Lalanne M., Lamrissi-Garcia I., Guyonnet-Duperat V., Richard E., Pitard V., et al. (2010) Modeling of congenital erythropoietic porphyria by RNA interference: a new tool for preclinical gene therapy evaluation. J Gene Med. 12, 637-646.

Varon C., Duriez A., Lehours P., Menard A., Laye S., Zerbib F., et al. (2009) Study of *Helicobacter pullorum* proinflammatory properties on human epithelial cells *in vitro*. Gut. 58, 629-635.

Varon C., Mocan I., Mihi B., Pere-Vedrenne C., Aboubacar A., Morate C., et al. (2014) *Helicobacter pullorum* cytolethal distending toxin targets vinculin and cortactin and triggers formation of lamellipodia in intestinal epithelial cells. J Infect Dis. 209, 588-599.
