# Supplementary figures and images for: The Cytolethal Distending Toxin Subunit CdtB of Helicobacter hepaticus Promotes Senescence and Endoreplication in Xenograft Mouse Models of Hepatic and Intestinal Cell Lines
Source: Front Cell Infect Microbiol. 2017 Jun 30;7:268. doi: 10.3389/fcimb.2017.00268 (PMC5491915; doi:10.3389/fcimb.2017.00268)

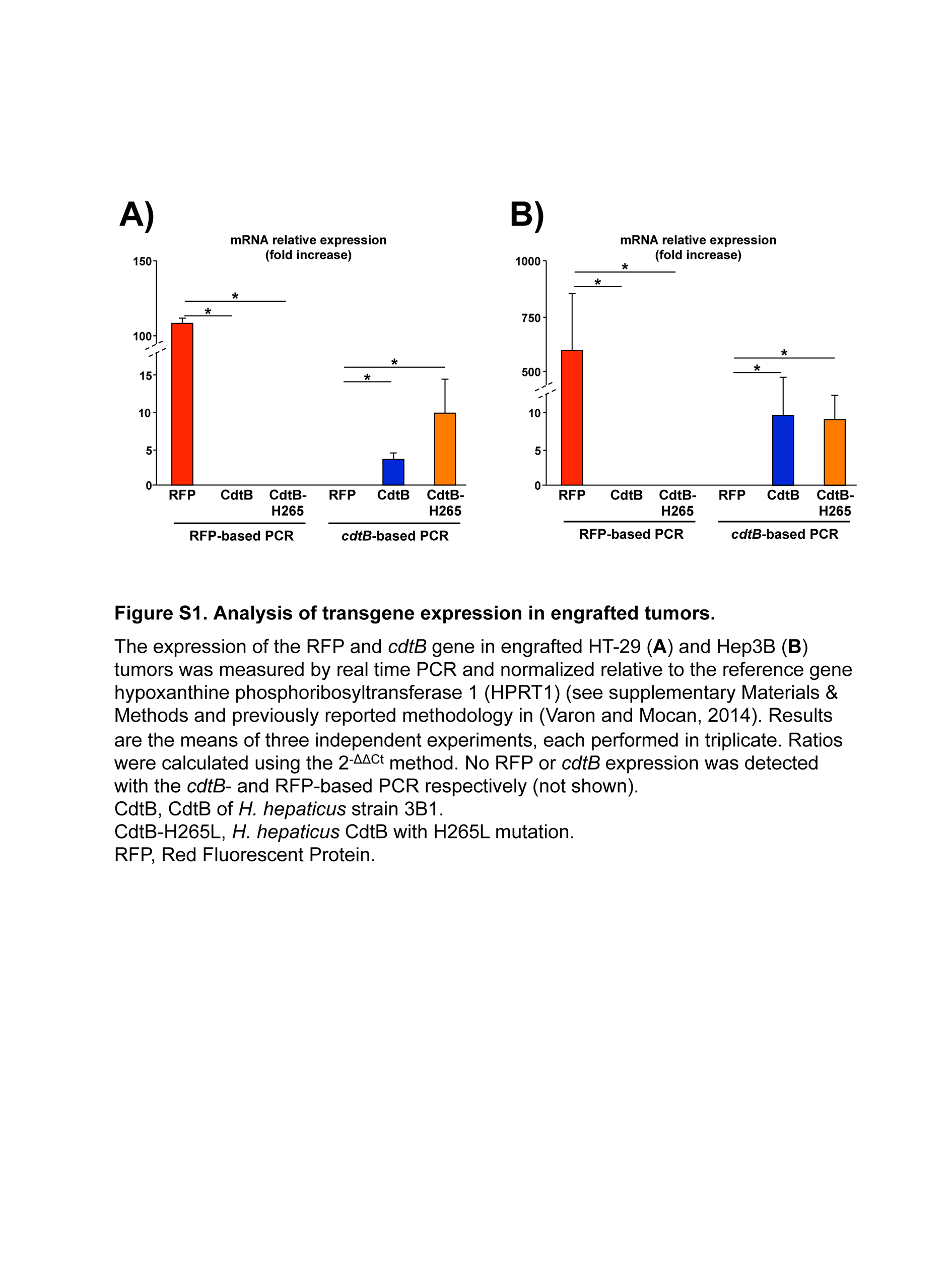

Supplement: Supplementary file 2 [file Image1.TIF]

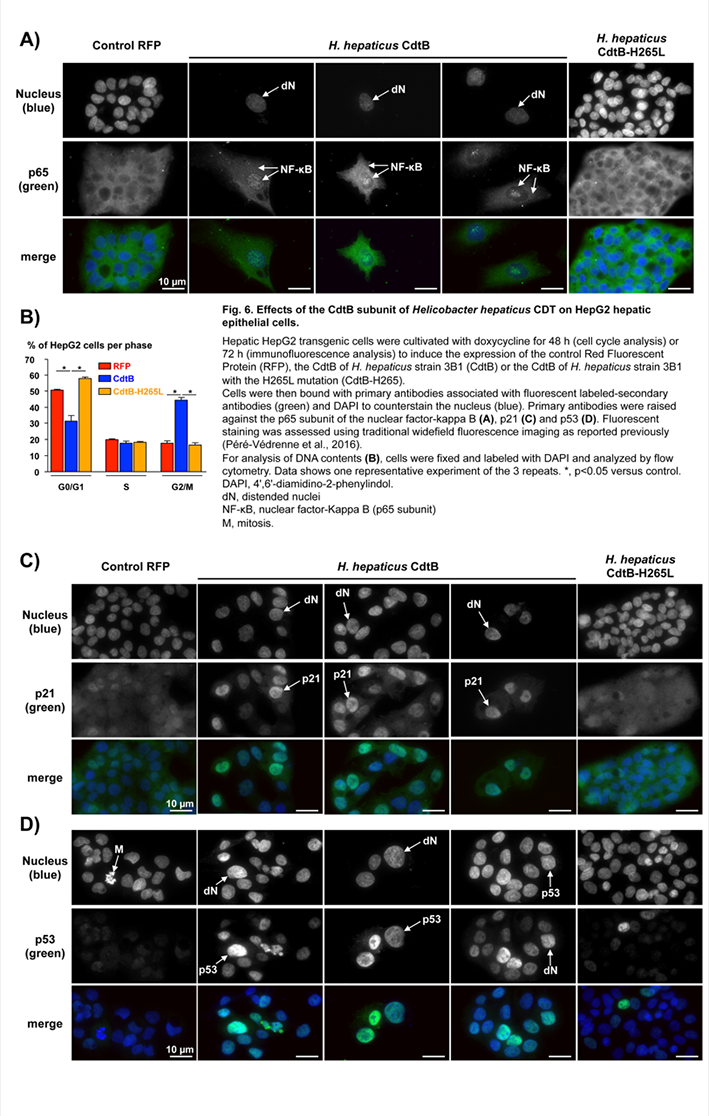

Supplement: Supplementary file 7 [file Image6.TIF]
