# Supplementary material for: The Cytolethal Distending Toxin Subunit CdtB of Helicobacter hepaticus Promotes Senescence and Endoreplication in Xenograft Mouse Models of Hepatic and Intestinal Cell Lines
Source: Front Cell Infect Microbiol. 2017 Jun 30;7:268. doi: 10.3389/fcimb.2017.00268 (PMC5491915; doi:10.3389/fcimb.2017.00268)
Supplement: Supplementary file 3 [file Image2.pdf]

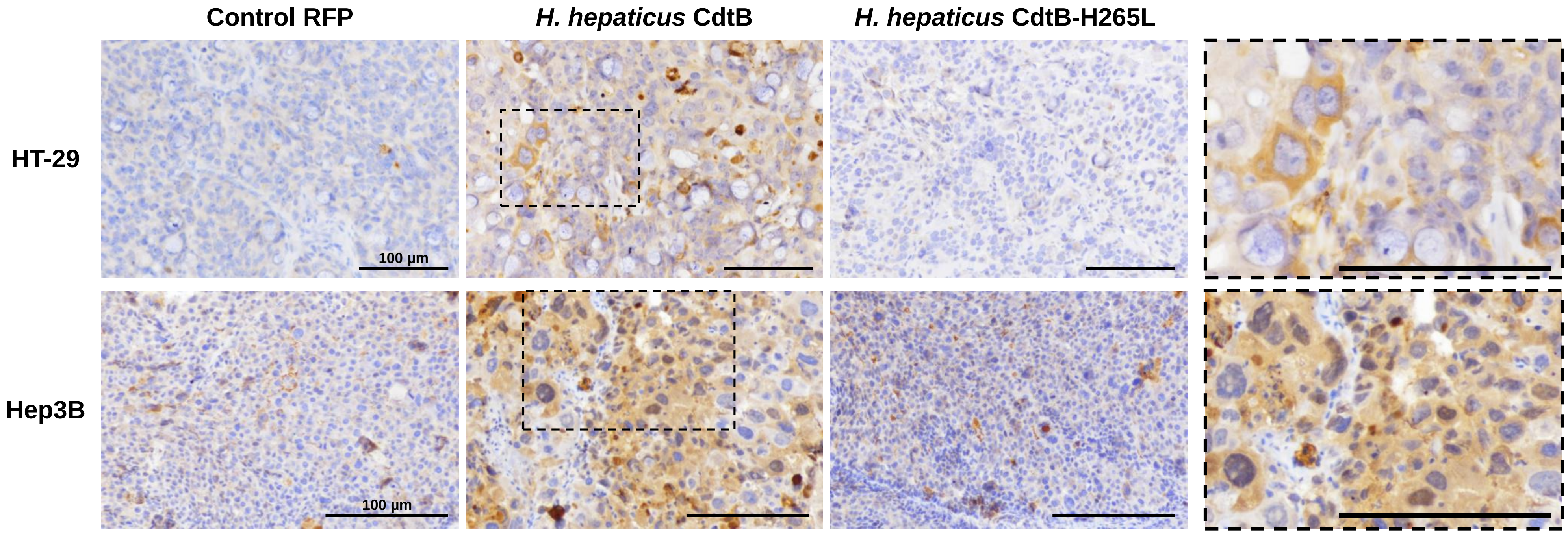

Fig. S2. Detection of the AKT protein phosphorylation (Ser473) in tumor xenografts.

Three µm-tissue sections of HT-29- and Hep3B-derived tumors were prepared from formalin-fixed paraffin-embedded tissues and submitted to standard hematoxylin staining and immunostaining raised against the phosphorylated AKT protein. Boxes correspond to enlargement.
Scale bars: 100 µm
CdtB, CdtB of *H. hepaticus* strain 3B1.
CdtB-H265L, *H. hepaticus* CdtB with H265L mutation.
RFP, red fluorescent protein.
