# Supplementary material for: The Cytolethal Distending Toxin Subunit CdtB of Helicobacter hepaticus Promotes Senescence and Endoreplication in Xenograft Mouse Models of Hepatic and Intestinal Cell Lines
Source: Front Cell Infect Microbiol. 2017 Jun 30;7:268. doi: 10.3389/fcimb.2017.00268 (PMC5491915; doi:10.3389/fcimb.2017.00268)
Supplement: Supplementary file 6 [file Image5.pdf]

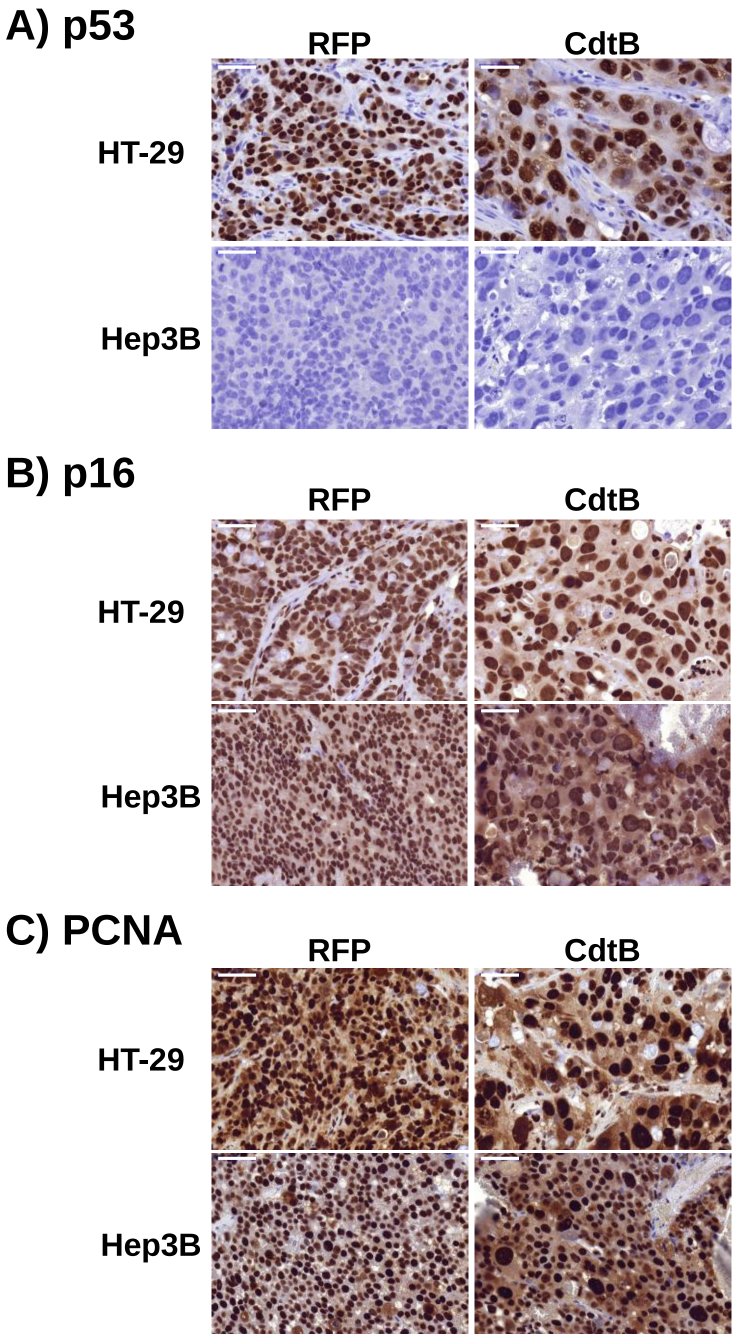

Figure S5. Effect of *Helicobacter hepaticus* CdtB expression in engraft tumors

After necropsy, 3 µm-tissue sections of HT-29- and Hep3B-derived tumors were prepared from formalin-fixed paraffin-embedded tissues and were subsequently submitted to standard hematoxylin staining and immunostaining raised against p53 **(A),** p16 **(B)** and proliferating cell nuclear antigen **(C)**. Scale bar, 50 µm.
CdtB, CdtB of *H. hepaticus* strain 3B1.
RFP, red fluorescent protein
